# Psychological resilience and functional recovery after acute ischemic stroke: a prospective cohort study

**DOI:** 10.3389/fneur.2026.1822458

**Published:** 2026-04-29

**Authors:** Ning Wang, Yaoyao Zhang

**Affiliations:** Cerebrovascular Disease Center, The affiliated Brain Hospital of Nanjing Medical University, Nanjing, Jiangsu, China

**Keywords:** acute ischemic stroke, Barthel Index, functional recovery, modified Rankin Scale, psychological resilience, stroke rehabilitation

## Abstract

**Background:**

Psychological resilience has been proposed as a factor that may influence recovery after stroke, yet evidence regarding its independent contribution to functional outcomes remains limited and inconsistent. This study evaluated the association between baseline psychological resilience and longitudinal functional recovery following acute ischemic stroke during structured rehabilitation.

**Methods:**

In this prospective cohort study, adult patients with imaging-confirmed acute ischemic stroke were enrolled during hospitalization and followed for 6 months. Psychological resilience was assessed using the 10-item Connor–Davidson Resilience Scale. Functional outcomes were measured using the modified Rankin Scale at 6 months and the Barthel Index at discharge, 3 months, and 6 months. Multivariable ordinal logistic regression was used to examine the association between resilience and functional outcome after adjusting for age, sex, stroke severity, comorbidity burden, rehabilitation exposure, and mood symptoms.

**Results:**

A total of 241 patients were included. Functional outcomes improved progressively over time, with the greatest gains observed during the early rehabilitation period. Baseline psychological resilience was associated with demographic and psychological characteristics but was not independently associated with functional recovery after adjustment for clinical factors. Sensitivity analyses using a binary definition of favorable outcome demonstrated an association between baseline stroke severity and recovery.

**Conclusion:**

In this prospective cohort of patients undergoing stroke rehabilitation, psychological resilience was not an independent predictor of functional outcome. Recovery was primarily determined by established clinical factors. These findings suggest that resilience may influence adaptation to illness rather than neurological recovery itself.

## Introduction

Stroke remains one of the leading causes of long-term disability and mortality worldwide, with ischemic stroke accounting for the majority of cases. Despite substantial advances in acute management and reperfusion therapies, a large proportion of survivors experience persistent functional limitations that affect independence, participation in daily activities, and overall quality of life. Recovery following acute cerebral infarction is a complex biological and behavioral process influenced by neurological injury, medical comorbidities, rehabilitation intensity, and psychosocial factors. Increasing attention has been directed toward psychological characteristics that may influence recovery trajectories, particularly the ability of patients to adapt to disability, remain engaged in rehabilitation, and maintain motivation during the recovery period. Beyond neurological injury itself, behavioral engagement during the rehabilitation phase plays a critical role in determining long-term recovery. Patients must actively participate in therapy, maintain motivation, and adapt psychologically to functional limitations. Consequently, psychological factors that influence coping and engagement have increasingly attracted attention as potential modifiers of rehabilitation outcomes. Psychological resilience has increasingly been recognized as a factor that may influence how patients cope with the challenges of acute illness and engage in the recovery process (1, 2).

Evidence from developed healthcare settings suggests that psychological resilience may play a meaningful role in recovery after major neurological injury. Studies conducted in the United States and several European countries have reported that stroke survivors with higher resilience demonstrate better emotional adjustment, improved participation in rehabilitation, and higher health-related quality of life during follow-up (3–5). However, findings across studies remain inconsistent. While some investigations report positive associations between resilience and post-stroke adjustment, others suggest that neurological severity and pre-stroke functional status remain the dominant determinants of physical recovery. Investigations from the United Kingdom and other Western populations have also suggested that resilience may buffer the negative impact of post-stroke depression and disability, potentially influencing functional outcomes. However, much of the existing literature has relied on cross-sectional designs or focused primarily on psychological well-being rather than objective functional recovery. In addition, several studies have not adequately accounted for rehabilitation exposure, mood disorders, or comorbidity burden, all of which may confound the relationship between psychological factors and recovery. In developing regions, available data remain limited. Studies from Asia have reported associations between resilience, mental health, and quality of life after stroke, yet few have examined whether resilience independently predicts functional recovery after adjusting for clinical severity and rehabilitation exposure (6–8). In China, where stroke represents a major public health burden, research has predominantly focused on biomedical predictors such as stroke severity, imaging findings, and vascular risk factors. Despite the high incidence of stroke and expanding rehabilitation services in China, prospective multidimensional studies integrating psychological characteristics with clinical predictors remain limited. Prospective multidimensional studies evaluating psychological resilience within the context of structured rehabilitation remain scarce, and the extent to which resilience contributes to functional recovery in this population has not been clearly established (9, 10).

Given these gaps in the literature, there is a need for prospective research integrating psychological and clinical determinants of recovery following acute cerebral infarction. Unlike previous studies that primarily evaluated psychological well-being, the present study integrates psychological resilience with detailed clinical, rehabilitation, and functional outcome data collected prospectively. Understanding whether resilience influences rehabilitation outcomes could provide new insights into patient-centered recovery and identify modifiable psychosocial targets for intervention. The present study was designed to investigate the role of psychological resilience in functional recovery among patients with acute ischemic stroke undergoing rehabilitation. Specifically, the present study aimed to examine the association between baseline psychological resilience and functional recovery after acute ischemic stroke, evaluate whether resilience modifies the relationship between stroke severity and outcome, and describe recovery trajectories during rehabilitation. By combining multidimensional clinical and psychological assessments within a prospective framework, this study seeks to clarify the contribution of resilience to post-stroke recovery and provide evidence that may inform individualized rehabilitation strategies.

## Materials and methods

### Study design and setting

This prospective longitudinal cohort study was conducted at the Cerebrovascular Disease Center of the Affiliated Brain Hospital of Nanjing Medical University. Patients were recruited between January 2023 and December 2024 and followed for 6 months after enrollment. The study aimed to evaluate the association between baseline psychological resilience and functional recovery following acute ischemic stroke during structured rehabilitation. Participants were followed from baseline assessment during hospitalization through discharge and post-discharge follow-up at 3 and 6 months.

### Participants

Consecutive adult patients aged 18 years or older with imaging-confirmed acute ischemic stroke were screened for eligibility. Patients were included if they were admitted within 7 days of symptom onset and were clinically stable to undergo neurological, functional, and questionnaire-based assessments.

Patients were excluded if they had severe cognitive impairment or aphasia preventing reliable questionnaire completion despite assistance, known psychotic disorders or severe psychiatric instability, terminal systemic illness with limited life expectancy, or anticipated inability to complete follow-up assessments. Pre-stroke functional status was recorded using the modified Rankin Scale and included as an adjustment variable in subsequent analyses rather than used as an exclusion criterion. The cohort primarily comprised patients who survived the acute hospitalization phase and were eligible for structured inpatient rehabilitation. Patients with malignant cerebral edema requiring palliative management or those who died during the hyperacute phase prior to rehabilitation assessment were not included in the longitudinal follow-up cohort.

### Data collection and baseline variables

Baseline demographic characteristics, vascular risk factors, and acute stroke treatments were obtained from medical records and patient or caregiver interview using standardized case report forms. Stroke severity at admission was assessed using the National Institutes of Health Stroke Scale (NIHSS). Comorbidity burden was evaluated using the Charlson Comorbidity Index. Pre-stroke functional status was recorded using the modified Rankin Scale.

Psychological resilience was assessed during early hospitalization using the 10-item Connor–Davidson Resilience Scale (CD-RISC-10). Depressive and anxiety symptoms were assessed using the Patient Health Questionnaire-9 (PHQ-9) and the Generalized Anxiety Disorder-7 (GAD-7) scale.

### Rehabilitation exposure

Rehabilitation exposure during hospitalization was quantified as the total minutes of physiotherapy, occupational therapy, and speech therapy received. Therapy dose was calculated as the cumulative therapy time during the inpatient rehabilitation period. Total rehabilitation exposure was modeled per 100-min increase to facilitate interpretation of effect estimates in regression analyses. Rehabilitation exposure was protocol-driven, with therapy delivered in standardized 30–45 min sessions according to institutional stroke rehabilitation pathways. Total therapy minutes represent cumulative delivered time recorded prospectively by treating therapists.

### Outcomes

The primary outcome was functional status at 6 months measured using the modified Rankin Scale (mRS). Deaths occurring during follow-up were coded as an mRS score of 6. Secondary outcomes included functional independence and longitudinal functional recovery measured using the Barthel Index at discharge, 3 months, and 6 months.

### Sample size considerations

Sample size considerations were based on supporting multivariable analysis of the primary outcome with prespecified covariates. Approximately 240–300 participants were targeted to ensure adequate statistical power and stability of regression estimates.

### Statistical analysis

Baseline characteristics were summarized using medians with interquartile ranges for continuous variables and counts with percentages for categorical variables. Group comparisons between resilience strata were performed using the Mann–Whitney *U* test for continuous variables and the chi-square test for categorical variables.

The association between baseline resilience and 6-month functional outcome was evaluated using multivariable ordinal logistic regression adjusting for age, sex, baseline NIHSS score, pre-stroke mRS, comorbidity burden, rehabilitation exposure, PHQ-9, and GAD-7. The proportional odds assumption was assessed prior to model interpretation. Interaction between resilience and baseline stroke severity was evaluated using a multiplicative interaction term.

Longitudinal changes in Barthel Index scores were evaluated using mixed-effects models including time and resilience group as fixed effects. Missing data were handled using multiple imputation where appropriate, with sensitivity analyses using complete-case models. Statistical significance was defined as a two-sided *p*-value <0.05. All analyses were performed using standard statistical software IBM SPSS Statistics version 20 (IBM Corp., Armonk, NY, United States). For the primary analysis, ordinal logistic regression was used to evaluate factors associated with 6-month modified Rankin Scale (mRS) outcome. The model estimated the odds of being in a lower (better) mRS category. The proportional odds assumption was assessed using the test of parallel lines and was not violated. Baseline NIHSS and other covariates were retained in the model irrespective of statistical significance based on established clinical relevance in stroke outcome prediction.

### Ethics statement

The study protocol was approved by the institutional research ethics committee of the Affiliated Brain Hospital of Nanjing Medical University. Written informed consent was obtained from all participants or their legally authorized representatives prior to enrollment. Data were de-identified and stored securely in accordance with institutional and national research guidelines.

## Results

### Study population

A total of 241 patients with acute ischemic stroke were included in the final analysis. The achieved sample size was consistent with the prespecified target for multivariable analysis. The median age of the cohort was 69 years (IQR 58–77), and 109 patients (45.2%) were male. The median National Institutes of Health Stroke Scale score at admission was 16 (IQR 12–25), indicating moderate-to-severe neurological impairment. The median baseline CD-RISC-10 score was 31 (IQR 26–34). Using the prespecified median split, 131 patients were categorized as low resilience and 110 as high resilience. At 6-month follow-up, 10 patients (4.1%) had died and deaths were incorporated into the ordinal outcome analysis as mRS score of 6, consistent with the predefined statistical approach.

### Baseline characteristics

Baseline demographic, clinical, and psychological characteristics are presented in Table 1. Age, sex distribution, and stroke severity were comparable between groups.

**Table 1:** Baseline characteristics.

Variable	Overall (*N* = 241)	Low resilience (*n* = 131)	High resilience (*n* = 110)	*p*-value
Age (years)	69 (58–77)	67 (56–75)	70 (59–77)	0.33
Male sex	109 (45.2%)	63 (48.1%)	46 (41.8%)	0.40
Baseline NIHSS	16 (12–25)	16 (12–26)	17 (12–24)	0.79
Pre-stroke mRS	0 (0–2)	0 (0–2)	0 (0–2)	0.62
Hypertension	124 (51.5%)	76 (58.0%)	48 (43.6%)	0.036
Diabetes mellitus	127 (52.7%)	81 (61.8%)	46 (41.8%)	0.003
Dyslipidemia	120 (49.8%)	67 (51.1%)	53 (48.2%)	0.74
Atrial fibrillation	70 (29.0%)	40 (30.5%)	30 (27.3%)	0.68
Previous stroke/TIA	69 (28.6%)	46 (35.1%)	23 (20.9%)	0.022
CD-RISC-10	31 (26–34)	26 (25–28)	35 (34–36)	<0.001
PHQ-9	11 (7–17)	12 (8–18)	10 (6–15)	0.37
GAD-7	6 (5–8)	6 (5–8)	6 (5–8)	0.68

Values represent median (IQR) or *n* (%).

NIHSS, National Institutes of Health Stroke Scale; mRS, Modified Rankin Scale, CD-RISC-10: 10-item Connor–Davidson Resilience Scale, PHQ-9, Patient Health Questionnaire-9, GAD-7, Generalized Anxiety Disorder-7.

However, vascular risk factors differed modestly between resilience strata. Hypertension was present in 58.0% of low-resilience patients compared with 43.6% in the high-resilience group (*p* = 0.036). Similarly, diabetes mellitus was more frequent in the low-resilience group (61.8% vs. 41.8%, *p* = 0.003), and prior stroke/TIA occurred in 35.1% versus 20.9% (*p* = 0.022).

Psychological variables demonstrated the expected separation between groups with significantly higher CD-RISC-10 scores in the high-resilience group (*p* < 0.001), whereas depressive and anxiety symptoms were similar. Overall comorbidity burden was moderate, with a median Charlson Comorbidity Index of 2 (IQR 1–3), with no significant difference between resilience groups.

### Rehabilitation exposure

Rehabilitation exposure during hospitalization was similar between groups (Table 2). Median inpatient rehabilitation duration was 6 days (IQR 5–8). The total therapy dose combining physiotherapy, occupational therapy, and speech therapy was 298 min (IQR 210–385) with no statistically significant difference between resilience strata. Post-discharge rehabilitation was received by 185 patients (76.8%).

**Table 2:** Rehabilitation exposure.

Variable	Overall (*N* = 241)	Low resilience (*n* = 131)	High resilience (*n* = 110)	*p*-value
Rehab days	6 (5–8)	6 (5–8)	7 (5–8)	0.20
Physiotherapy minutes	105 (88–140)	105 (88–140)	114 (88–140)	0.08
Occupational therapy minutes	105 (88–140)	105 (88–140)	105 (88–140)	0.29
Speech therapy minutes	0 (0–122)	0 (0–122)	35 (0–122)	0.21
Total therapy minutes	298 (210–385)	280 (184–385)	315 (228–402)	0.15
Missed sessions	0 (0–1)	0 (0–1)	0 (0–1)	0.99
Post-discharge rehab	185 (76.8%)	103 (78.6%)	82 (74.5%)	0.55

Values are presented as median (interquartile range) or number (percentage).

Total therapy minutes represent the cumulative time of physiotherapy, occupational therapy, and speech therapy received during hospitalization.

### Functional outcomes

Functional recovery improved substantially over time. Median mRS improved from 3 at discharge to 2 at 6 months, while Barthel Index increased from 58 to 83. A difference between groups was observed at discharge (*p* = 0.007), but this difference disappeared during follow-up. Functional recovery was most pronounced during the early post-stroke period, with the greatest improvement observed between discharge and the 3-month follow-up. Although median mRS remained 3 at discharge and 3 months, distribution analysis demonstrated a shift toward lower disability categories over time, with further improvement evident by 6 months. Median values alone may underestimate incremental category transitions in ordinal functional scales (see Table 3).

**Table 3:** Functional outcomes.

Outcome	Overall	Low resilience (*n* = 131)	High resilience (*n* = 110)	*p*-value
mRS discharge	3 (2–3)	3 (3–3)	3 (2–3)	0.007
mRS 3 months	3 (2–3)	3 (2–3)	3 (2–3)	0.11
mRS 6 months	2 (2–3)	2 (2–3)	2 (2–3)	0.90
Barthel discharge	58 (54–64)	58 (56–64)	58 (54–64)	0.71
Barthel 3 months	75 (69–78)	76 (70–78)	74 (69–79)	0.51
Barthel 6 months	83 (79–89)	84 (79–88)	83 (80–89)	0.93
Mortality	10 (4.1%)	4 (3.1%)	6 (5.5%)	0.54

Values are presented as median (interquartile range) or number (percentage).

Lower mRS scores indicate better functional outcome.

Higher Barthel Index scores indicate greater functional independence.

### Primary multivariable analysis

In the adjusted ordinal logistic regression model evaluating predictors of 6-month disability, baseline psychological resilience was not independently associated with outcome (see Table 4).

**Table 4:** Multivariable ordinal logistic regression.

Variable	Adjusted OR	95% CI	*p*-value
Age (per year)	0.97	0.95–0.99	0.021
Male sex	1.38	0.86–2.22	0.180
Baseline NIHSS (per 1-point increase)	0.93	0.89–0.97	0.002
Pre-stroke mRS	0.89	0.63–1.26	0.510
Charlson Comorbidity Index	0.94	0.82–1.09	0.430
Rehabilitation dose (per 100-min increase)	1.06	0.98–1.15	0.140
PHQ-9	0.98	0.92–1.05	0.590
GAD-7	1.01	0.97–1.04	0.640
CD-RISC-10 (per point)	1.02	0.96–1.08	0.480

OR >1 indicates higher odds of better (lower) mRS category. OR <1 indicates lower odds of favorable outcome. NIHSS: National Institutes of Health Stroke Scale; mRS: modified Rankin Scale; CCI, Charlson Comorbidity Index; CD-RISC-10, Connor–Davidson Resilience Scale-10; PHQ-9, Patient Health Questionnaire-9; GAD-7, Generalized Anxiety Disorder-7.

Baseline NIHSS score remained a significant predictor of functional status. The proportional odds assumption was not violated. The interaction between psychological resilience and baseline stroke severity was not statistically significant (*p* = 0.234), indicating that resilience did not modify the relationship between stroke severity and functional outcome.

**Figure 1:**
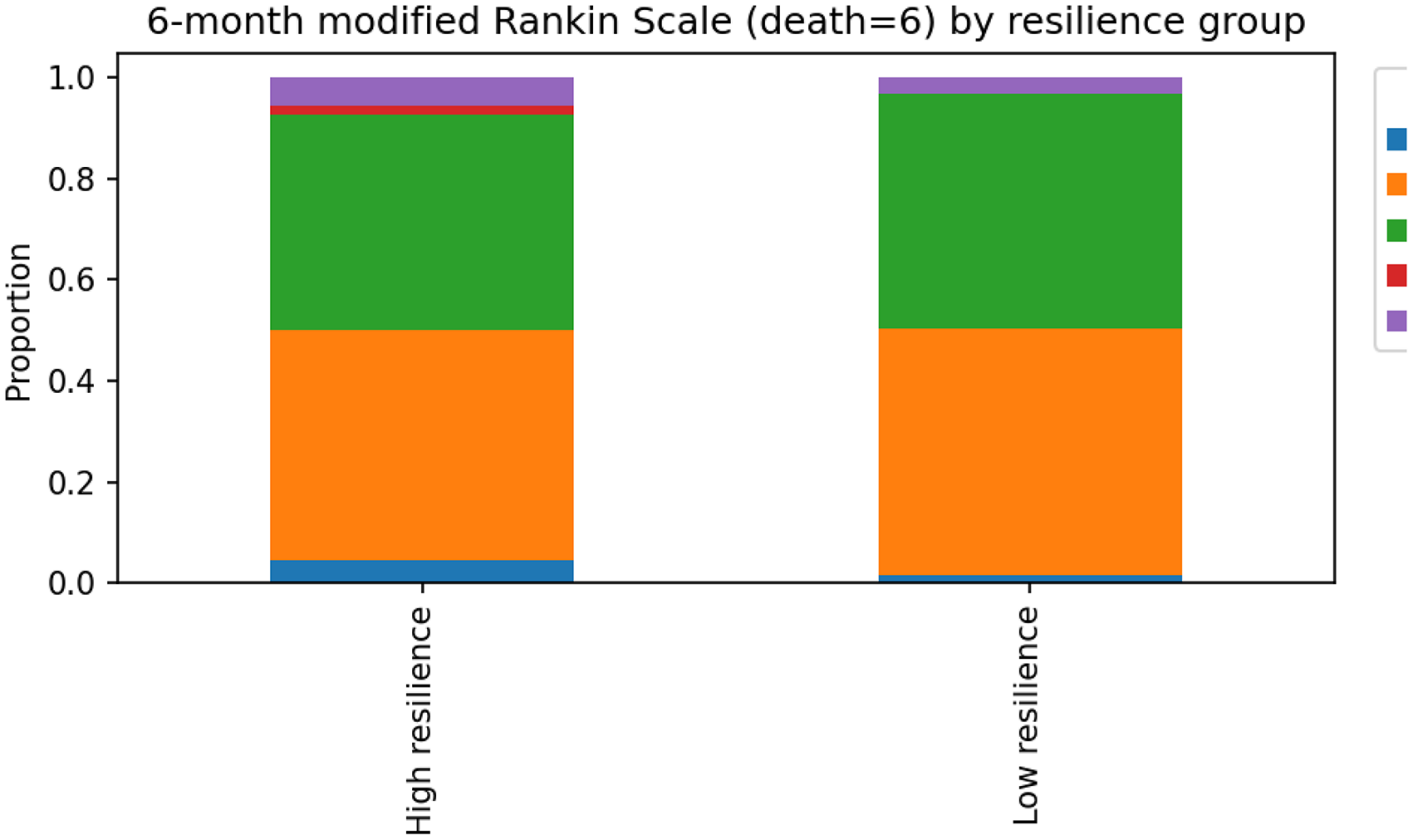
Distribution of 6-month modified Rankin Scale scores. Bar chart showing the distribution of disability levels at 6-month follow-up among patients with acute ischemic stroke. Lower scores indicate better functional outcomes, whereas higher scores represent greater disability or death. Most patients demonstrated improvement compared with discharge status, with the majority achieving moderate disability levels at follow-up.

**Figure 2:**
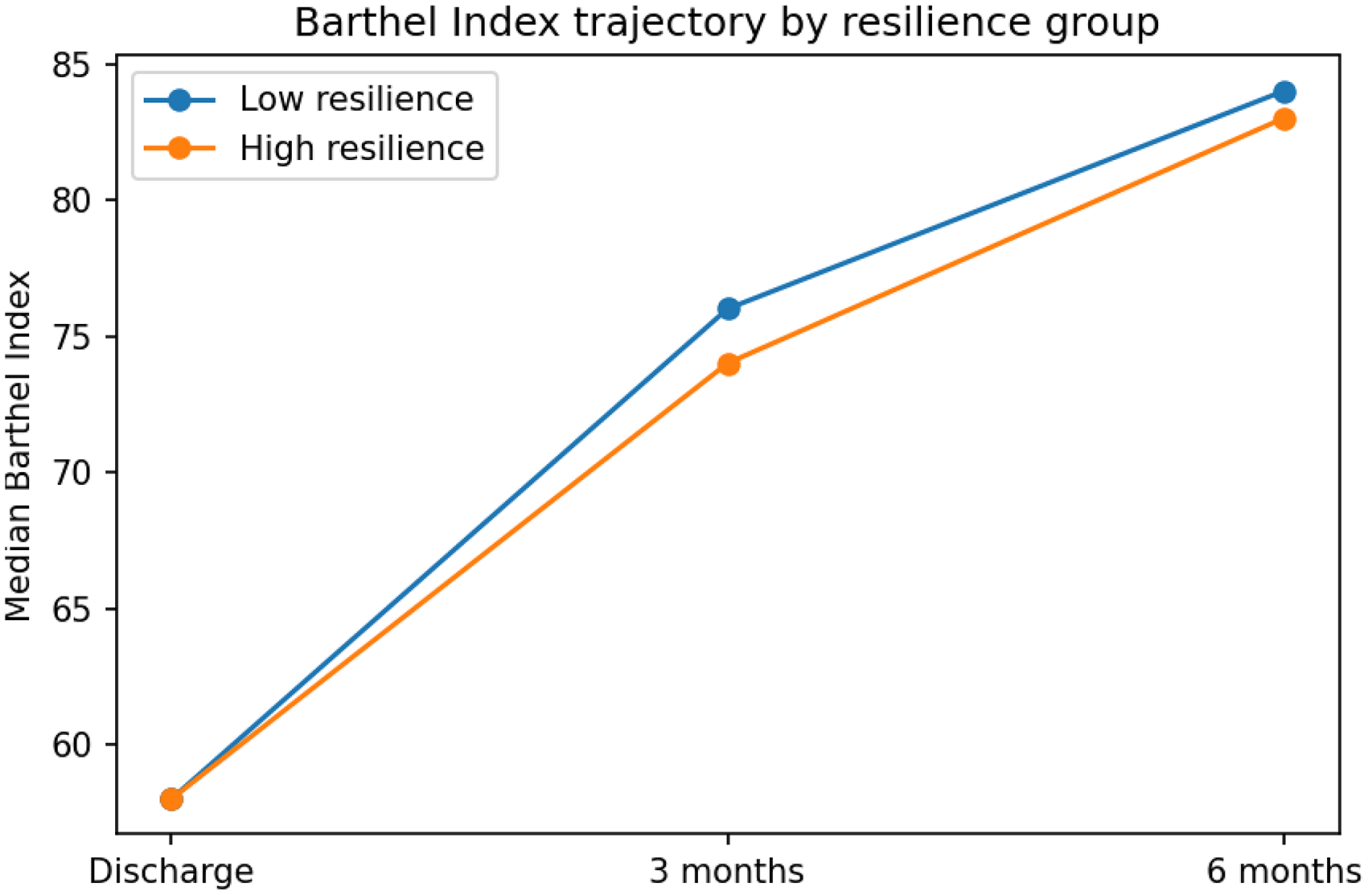
Change in Barthel Index over time. Line graph illustrating functional recovery measured by the Barthel Index at discharge, 3 months, and 6 months. Scores increased progressively during follow-up, with the largest improvement observed between discharge and the 3-month assessment, indicating substantial recovery during the early rehabilitation phase.

In sensitivity analyses using a binary definition of favorable outcome (mRS ≤ 2), baseline stroke severity was associated with outcome. Rehabilitation dose was not significantly associated with functional outcome after adjustment. Adjustment for overall comorbidity burden using the Charlson Comorbidity Index did not materially change the association between resilience and functional outcome (see Table 5, Figures 1, 2).

**Table 5:** Multivariable logistic regression for favorable outcome (mRS ≤ 2).

Variable	Adjusted OR	95% CI	*p*-value
Age (per year)	0.97	0.94–1.00	0.067
Male sex	1.41	0.83–2.40	0.198
Baseline NIHSS	0.95	0.91–0.99	0.018
Pre-stroke mRS	0.88	0.59–1.32	0.540
CCI	0.91	0.76–1.09	0.300
Rehabilitation dose (per 100 min)	1.07	0.94–1.21	0.310
PHQ-9	0.98	0.90–1.07	0.660
GAD-7	1.01	0.98–1.03	0.490
CD-RISC-10	1.02	0.96–1.09	0.470

## Discussion

The present prospective cohort study evaluated whether baseline psychological resilience influences functional recovery following acute ischemic stroke and whether resilience modifies the relationship between stroke severity and outcome during rehabilitation. In this cohort, functional outcomes improved progressively during follow-up, particularly in the early rehabilitation period. However, baseline psychological resilience was not independently associated with functional recovery after adjustment for clinical factors. Instead, established predictors such as stroke severity and baseline health status remained the primary determinants of outcome. These findings suggest that although resilience reflects important psychological characteristics, its independent contribution to neurological recovery after stroke may be limited once biological and clinical variables are considered. Although baseline stroke severity remains a well-established determinant of functional outcome, resilience did not retain independent prognostic significance after adjustment for NIHSS and other clinical covariates. This suggests resilience may be more relevant to psychological adjustment and engagement rather than directly determining neurological functional recovery.

Previous research has suggested that psychological resilience may influence recovery experiences following major illness and disability. Studies have reported that individuals with higher resilience tend to demonstrate better psychological adaptation, improved quality of life, and greater engagement with rehabilitation following neurological injury (11–14). Resilient individuals may utilize more effective coping strategies, maintain motivation during therapy, and demonstrate better emotional regulation, which could indirectly support recovery. Additionally, resilience has been proposed as a modifiable characteristic that may enhance participation in rehabilitation and long-term adjustment following stroke (15, 16). These mechanisms provide a plausible explanation for why resilience has attracted increasing interest in stroke recovery research.

Despite these theoretical benefits, the present findings did not demonstrate an independent association between resilience and functional outcome after adjustment for clinical variables. This observation is consistent with several prior investigations indicating that stroke recovery is predominantly influenced by neurological injury severity, age, pre-existing disability, and comorbidity burden (17–19). Large cohort studies have shown that baseline stroke severity measured by the NIH Stroke Scale is one of the strongest predictors of long-term outcome (20). Similarly, systematic reviews have reported that while psychosocial variables may affect subjective well-being, their effect on objective functional measures such as the modified Rankin Scale is often modest after accounting for clinical factors (21). These findings support the interpretation that resilience may play a supportive rather than primary role in determining recovery.

Another possible explanation relates to the characteristics of the study population. Patients in this cohort demonstrated moderate-to-severe neurological impairment at baseline, which may limit the potential influence of psychological variables on long-term outcomes. In populations with more severe strokes, biological injury often dominates recovery trajectories, potentially overshadowing the contribution of psychosocial characteristics (22). Furthermore, resilience may exert indirect effects through pathways such as mood regulation, adherence to therapy, or social engagement, which are not fully captured by traditional functional outcome measures. It is therefore possible that resilience influences aspects of recovery not directly reflected in measures such as the modified Rankin Scale or Barthel Index (23).

The present study has several strengths. The prospective design allowed systematic evaluation of psychological resilience early in the course of stroke, and patients were followed longitudinally with validated functional outcome measures. The analysis also accounted for multiple clinically relevant variables including stroke severity, pre-stroke disability, comorbidity burden, and rehabilitation exposure. This comprehensive approach strengthens the validity of the findings and provides a balanced assessment of psychological and clinical determinants of recovery.

Several limitations should also be acknowledged. First, the study was conducted at a single center, which may limit generalizability to other populations or healthcare systems. Second, resilience was measured at a single time point, whereas psychological responses may change during recovery. Third, unmeasured factors such as social support, caregiver involvement, or socioeconomic status could influence outcomes and were not fully captured in the present analysis. Finally, although the sample size was adequate for multivariable analysis, larger multicenter studies may be better positioned to detect smaller associations between psychological factors and stroke recovery.

Future research should further explore how resilience interacts with mood disorders, rehabilitation engagement, and social factors throughout the recovery process. Longitudinal studies incorporating repeated psychological assessments may provide greater insight into whether resilience evolves during rehabilitation and whether such changes influence outcomes. Interventions aimed at enhancing resilience and coping strategies may also help improve patient-reported outcomes and quality of life following stroke.

## Conclusion

In this prospective cohort of patients undergoing rehabilitation after acute ischemic stroke, functional recovery improved over time, particularly during the early post-stroke period. However, baseline psychological resilience was not independently associated with functional outcome after adjustment for established clinical predictors. Stroke severity and baseline health status remained the primary determinants of recovery. These findings suggest that psychological resilience may influence aspects of adaptation to illness but does not appear to independently determine neurological recovery following stroke.

## Data Availability

The datasets presented in this article are not readily available because access to the dataset is restricted due to institutional ethics regulations and patient confidentiality policies. Requests to access the datasets should be directed to zyaoyao683@gmail.com.
